# Together Apart: The Mitigating Role of Digital Communication Technologies on Negative Affect During the COVID-19 Outbreak in Italy

**DOI:** 10.3389/fpsyg.2020.554678

**Published:** 2020-10-21

**Authors:** Alessandro Gabbiadini, Cristina Baldissarri, Federica Durante, Roberta Rosa Valtorta, Maria De Rosa, Marcello Gallucci

**Affiliations:** ^1^University of Milano-Bicocca, Department of Psychology – Mind and Behavior Technological Center, Milan, Italy; ^2^University of Milano-Bicocca, Department of Psychology, Milan, Italy

**Keywords:** COVID-19, social isolation, social support, digital technology, negative affect

## Abstract

The ongoing pandemic of COVID-19 has forced governments to impose a lockdown, and many people have suddenly found themselves having to reduce their social relations drastically. Given the exceptional nature of similar situations, only a few studies have investigated the negative psychological effects of forced social isolation and how they can be mitigated in a real context. In the present study, we investigated whether the amount of digital communication technology use for virtual meetings (i.e., voice and video calls, online board games and multiplayer video games, or watching movies in party mode) during the lockdown promoted the perception of social support, which in itself mitigated the psychological effects of the lockdown in Italy. Data were collected in March 2020 (*N* = 465), during the lockdown imposed to reduce the COVID-19 spread. The results indicated that the amount of digital technology use reduced feelings of loneliness, anger/irritability, and boredom and increased belongingness *via* the perception of social support. The present study supported the positive role of digital technologies in maintaining meaningful social relationships even during an extreme situation such as a lockdown. Implications such as the need to reduce the digital divide and possible consequences of the ongoing pandemic are discussed.

## Introduction

A worldwide outbreak of severe acute respiratory syndrome (SARS)-CoV-2 (COVID-19) begun in December 2019. At the beginning of April 2020, it caused more than 138,000 deaths and had infected 2 million individuals worldwide (World Health Organization, 2020a). With more than 40,000 recorded infections, Italy was considered the second epicenter of the pandemic (Horowitz et al., 2020). As the number of infected people continued to rise, the Italian government enacted a decree on March 8, 2020 (OJ-N.59 of 8-3-2020), imposing a lockdown to the whole country, aimed at preventing the spread of the virus. The term *lockdown* refers to stringent containment measures, such as quarantine and social distancing, in order to slow down the spread of COVID-19. At the beginning of April 2020, 165,000 Italians had been infected, and among these, 62,000 were in Lombardy (Opendata, 2020), one of the regions most affected by the infection.

Potential negative feelings that people can experience in the current emergency period, such as anxiety, loneliness, boredom, anger, and irritability, have been recently listed by both the WHO and the APA (American Psychological Association, 2020; World Health Organization, 2020b).

Because of the restrictions imposed during the ongoing pandemic, populations have been asked to reduce social relations. Therefore, in the present study, we wanted to investigate the role of modern digital communication technologies in facilitating the maintenance of meaningful social relationships and promoting the perception of social support. We also examined whether the perception of social support due to the use of technologies for virtual meetings mitigated some of the possible negative psychological states during the pandemic.

## The Role of Perceived Social Support During a Lockdown

Despite the need to reduce the contagion, social isolation can have several psychological consequences, leading to post-traumatic stress symptoms (Brooks et al., 2020). Among the stressors, there are infection fears, frustration, boredom, inadequate supplies and information, financial loss, stigma, and longer isolation duration (Brooks et al., 2020). Social isolation also collides with the fundamental needs of belongingness, the human emotional need to give and receive attention from others (Baumeister and Leary, 1995; Fiske, 2018). Indeed, the depth of connection people have with significant others is one of the constituents of psychological well-being (Ryff, 2014), by promoting a greater sense of self-worth and belongingness (Oh et al., 2014). On the contrary, the perception of lacking social support is strictly associated with loneliness (Lin et al., 2020), irritability and anger (e.g., Arslan, 2009), boredom and depression (Gariepy et al., 2016), and anxiety (e.g., Wang et al., 2018). In this regard, research consistently demonstrates that the perceived availability of social support, intended as a real or perceived experience that one is cared for and part of a mutually supportive social network (Taylor, 2011), reduces psychological distress, providing resources that can weaken the negative consequences of acute stressors (Cohen and McKay, 1984; Cohen and Wills, 1985; Lakey and Cohen, 2000). Therefore, social support could represent a relevant protective factor for mitigating the overall negative psychological consequences, supporting psychological well-being during a lockdown.

## During a Lockdown, Everyone Wants to Be—Virtually—Connected

One substantial difference between the current COVID-19 pandemic and previous epidemics is the amount of tech tools that we can use today compared to the past. The technology available nowadays allows people to stay in contact with others in innovative ways, from virtual happy hours with friends to religious services. The worldwide increased use of online tools during the coronavirus lockdown has been registered by all the main digital platforms and social media (Perez, 2020). A recent review of studies (Waytz and Gray, 2018) suggests that online technology for communication may function both as a social connector and a separator. On the one hand, online communication reduces the social cues typical of face-to-face interactions, encouraging more impersonal interactions (White and Dorman, 2001) and making exchanging support more difficult (Lewandowski et al., 2011). Online communications have also been found to be associated with decreased empathy (Konrath et al., 2011) and increased individualism (Wellman et al., 2003). On the other hand, technology helps in maintaining social connections *via* digital communication platforms (Genoe et al., 2018), providing support for people for whom face-to-face social interactions are difficult to obtain (e.g., Fogel et al., 2002; Barak and Sadovsky, 2008; Delello and McWhorter, 2015). Waytz and Gray (2018) suggest that, depending on different factors such as age, generation, and developmental differences in technology use, online communication can improve social relationships when people use it to strengthen existing relationships with off-line friends and family, especially when in-person social interactions are impeded by external factors, such as a lockdown.

Indeed, the exceptionality of the restrictions imposed by the COVID-19 pandemic represented a unique situation for testing on the field the functioning of digital technologies for communication and virtual meetings as a substitute for meaningful face-to-face relationships. Indeed, the adoption of communication technologies during a lockdown could play a key role in favoring the perception of social support, which in turn could function as a buffer against the negative impact of social restrictions.

## The Study

The main aim of the present study was to verify whether online tech tools for communication and virtual meetings could reduce the negative psychological consequences of a lockdown. To do so, we tested the hypothesis that the amount of online communication usage (i.e., video calls, online board games, and streaming movie in party mode) during the lockdown that occurred in Italy would be positively associated with the perception of social support. In this case, the latter would be negatively related to loneliness, irritability, boredom, anger, and anxiety and positively associated with belongingness.

## Method

### Participants

For the sake of reliability, we intended to collect data on a large scale (i.e., *N* > 250). This guarantees high power for small and medium correlations (power = 0.95) and stability of correlations (Schönbrodt and Perugini, 2013).

In total, 899 participants accessed the online survey: 106 participants did not consent to participate in the study, 39 did not give the final consent for the data processing, eight did not indicate whether they are of legal age, 20 declared that they were not of legal age, and 80 participants did not fill in any data and were considered as dropouts. In order to monitor the level of participants’ attention, we included two “catch-trials” in our survey (i.e., “Please answer 2 to this item” and “Please answer 6 to this item”; see Oppenheimer et al., 2009; Leys et al., 2018): 180 respondents failed one or both items. Finally, one participant presented missing data.

All these participants were excluded from the final sample, which therefore consisted of 465 respondents (completion rate, 51.7%; 348 females, 116 males, one preferring not to answer, min age = 18 years, max age = 73 years, mean age = 31.29 years, SD = 13.19). Based on a Monte Carlo power analysis for mediation model, ran with 20.000 Monte Carlo Draws with 1,000 replications and a 95% confidence level, a sample of 465 participants guarantees a power of 0.98 for small indirect effects (IE = 0.20) and of 0.99 for medium effects (IE = 0.50; see also Perugini et al., 2018).

Overall, 72.5% of the participants in the final sample reside in Lombardy—the Italian region most affected by the virus—and the remaining 27.5% in the rest of Italy.

### Procedures

Data were collected through a questionnaire using Qualtrics web system between March 20 and April 2, 2020. The data collection started about 2 weeks from the beginning of the lockdown that the Italian Government adopted for the urgent containment and management of the COVID-19 epidemiological emergency. By adopting a snowball sampling technique, the participants were recruited through social media and instant messaging systems, by sending a link to the web survey, and by asking to forward the link to their contacts.

### Measures

#### Amount of Technology Use

We asked the participants to report how many times they had used different tools to stay connected during the lockdown prior to the data collection. Using six items, the participants were asked to report how many times they: (1) made or received a video call for a virtual dinner or lunch with their friends, partner, and/or family; (2) made or received a video call for a leisure meeting with their friends, their partner, and/or family; (3) made or received a voice call with their friends, partner, and/or family; (4) watched a movie in party mode; (5) played online board games with their friends, partner, and/or family; and (6) played multiplayer online video games. All frequency items were measured on the following scale: 1 = never, 2 = about once a week, 3 = from one to three times a week, 4 = from four to six times a week, 5 = once a day, and 6 = several times a day. The scores reported were then averaged to obtain an overall index of technology usage during the lockdown.

Since the same technologies could also be used for work (e.g., virtual meeting) and school (e.g., online streaming lectures), we asked the participants to report the frequency with which they (1) made or received a video call for work/school and (2) made or received a voice call for work/school. Both items were measured on the same response scale illustrated above. The scores were then averaged as an overall index of technology use for work/school activities.

The following measures were then used to assess participants’ emotional state during the lockdown. Scales were presented in a random order to prevent response bias and were introduced with the following instruction: “Please, respond to the following statements thinking about how you felt during the last weeks of lockdown.”

#### Perceived Social Support

We adapted the Multidimensional Scale of Perceived Social Support (Zimet et al., 1988), composed of 12 items identifying different sources of social support. Sample items are “I get the emotional help and support I need from my family” and “My friends really try to help me” (1 = strongly disagree to 7 = strongly agree).

#### Loneliness

We used the UCLA Loneliness Scale-Revised (Russell et al., 1980), a 20-item scale designed to measure subjective feelings of loneliness and social isolation. Sample items are “I have nobody to talk to” and “I feel left out” (1 = I never feel this way to 7 = I always feel this way).

#### State Irritability

We used the Brief Irritability Test (Holtzman et al., 2015), composed of five items in which the participants are asked to indicate how frequently they identify with each statement. Sample items are “I have been feeling irritable” and “Things have been bothering me more than they normally do” (1 = never to 7 = always).

#### State Boredom

We adopted the Italian version of the Multidimensional State Boredom Scale (MSBS; Fahlman et al., 2011; Craparo et al., 2017). The scale consists of 29 items assessing an individual’s experience of state boredom. Sample items are “I feel bored” and “Time is passing by slower than usual” (1 = completely disagree to 7 = completely agree).

#### State Anger

The State-Trait Anger Expression Inventory (Forgays et al., 1997), composed of 10 items, was employed to assess participants’ intensity of anger as an emotional state. Sample items are “I feel angry” and “I feel like swearing” (1 = completely disagree to 7 = completely agree).

#### State Anxiety

We used the short-form of the State-Trait Anxiety Inventory (Spielberger et al., 1983; Marteau and Bekker, 1992), composed of six items (e.g., “I feel nervous” and “I feel worried”; 1 = completely disagree to 7 = completely agree).

#### Belongingness

We used two five-part items adapted from McFarland et al. (2012), asking how close and how often participants use the word “we” to refer to several groups (e.g., family/friends/people in their community/Italians/people all over the world; 1 = never, 7 = very often).

#### Demographics and Control Measures

Previous literature suggested that negative affective states could vary as a function of time (Rubins, 1964). Since social distancing measures had been amended several times by the Italian government, after providing demographic data, the participants were asked to report the actual number of days they had already spent in isolation. Besides, the regulations provided some exceptions, such as going to work (only for specific categories of workers) and shopping for essential goods (e.g., food and pharmaceuticals). Therefore, we asked the participants to report the number of house exits that were made during the lockdown period (1 = never, 2 = about once a week, 3 = from one to three times a week, 4 = from four to six times a week, 5 = once a day, 6 = several times a day).

The forced isolation imposed by the lockdown could be harsher for people living alone or sharing confined spaces. The related literature suggests that several situational factors can be related to negative affect (Zysberg, 2015), such as the number of people living with (Savikko et al., 2005), living arrangements, and housing type (Krause-Parello and Gulick, 2013). Therefore, we asked the participants to report how many people they lived with during the lockdown and report their home/apartment size.

Finally, as control variables, we asked the participants to estimate their frequency of social technology usage to maintain social relationships and work/school motives before the lockdown period. To do so, the items created for assessing the overall amount of technology use during the lockdown were adapted by asking the participants to report the frequency of technology usage for social connections and business/school purposes by referring to their everyday life before the lockdown. The obtained scores were then averaged to create two separate indexes for the amount of technology use for maintaining social relationships and work/school motives before the lockdown.

## Results

### Preliminary Analyses

Before conducting the analyses, data were inspected for normality and outliers. Separate multiple and simple regression models were tested considering the amount of technology use during the lockdown and social support as the predicting variables, whereas loneliness, boredom, anxiety, anger, irritability, and belongingness were entered as outcomes. Standardized residuals, skewness, and kurtosis values were all < 1.0, indicating a normal distribution of the residuals (Bulmer, 1979). Outliers were inspected by plotting Cook’s distances by centered leverage values of the residuals for each regression model (Cook, 1977). Two influential data points emerged as common outliers in most of the tested models. Therefore, they were excluded from all the subsequent analyses and all the analyses performed on a sample of *N* = 463 (see also Supplementary Material).

At the time of the data collection, the participants reported having already spent about 14 days in isolation; 44 participants reported having left home for work reasons and 323 having left home for buying food. These two indicators were summed as an overall index of exits made during the lockdown period. On average, participants left home between one and three times a week.

Regarding the housing situation, 41 individuals stated that they were living alone and 422 with their family, flat mates, or their partner. On average, a family unit is composed of three people, and the average size of the houses/apartments was around 123 m^2^.

To verify whether participants reported different levels of use of technologies for maintaining their social relationships during the lockdown compared to the past, a series of *t*-tests were performed. The results (see Table 1) highlighted a significant increase in the use of all technologies, except for voice calls for work/school.

**TABLE 1:** Mean comparisons for the frequency of technology use before and during the lockdown.

Use of technologies	Mean pre-lockdown (SD)	Mean during lockdown (SD)	Cohen’s *d*	*t* Test
Video calls for virtual dinner/lunch	1.15 (0.57)	1.67 (1.08)	0.45	*t*(462) = 9.77, *p* < 0.001
Video calls for leisure meeting	1.46 (0.98)	3.05 (1.58)	0.93	*t*(462) = 19.93, *p* < 0.001
Streaming movies in party mode	1.57 (1.13)	1.88 (1.56)	0.27	*t*(462) = 6.07, *p* < 0.001
Online board games	1.46 (1.15)	2.07 (1.65)	0.52	*t*(462) = 11.22, *p* < 0.001
Multiplayer online video games	1.33 (0.97)	1.57 (1.32)	0.27	*t*(462) = 5.87, *p* < 0.001
Making or receiving voice calls from friends, partner, and family	3.56 (1.68)	4.28 (1.53)	0.48	*t*(462) = 10.44, *p* < 0.001
Making or receiving voice calls for work/school	2.56 (1.92)	2.37 (1.80)	0.13	*t*(462) = −2.73, *p* = 0.007
Making or receiving video calls for work/school	1.30 (0.91)	2.43 (1.62)	0.69	*t*(462) = 14.77, *p* < 0.001

**N* = 463.*

Participants reported increased use of digital communication technologies during the lockdown compared to the past. They watched more streaming movies in party mode and played more online board games with their friends and multiplayer online video games compared to the period before the lockdown. The use of voice calls also increased, with participants reporting to have made or received more voice and video calls from their friends, partner, and/or family, but less voice calls for business/school motives than the pre-lockdown period.

Cronbach’s alphas were ≥ 0.80 for all scales (see Table 2). Given the adequate internal consistency, we calculated composite scores for each scale, and correlational analysis was performed on all our variables. Table 2 summarizes these results.

**TABLE 2:** Descriptive statistics and correlations among variables.

		***α***	***M***	**SD**	**1**	**2**	**3**	**4**	**5**	**6**	**7**	**8**	**9**	**10**	**11**	**12**	**13**	**14**	**15**	**16**
1	Age	−	31.26	13.19																
2	Gender	−	−	−	−0.163**															
3	Days of isolation	−	14.15	7.18	−0.206**	0.075														
4	Number of exits	−	2.36	1.63	0.358**	–0.089	−0.550**													
5	Number of persons living with	−	2.96	1.30	−0.283**	0.027	0.075	−0.118*												
6	House sqm	−	123.09	77.09	−0.106*	0.002	0.085	–0.055	0.357**											
7	Past technology use	−	1.75	0.55	0.168**	−0.149**	–0.013	0.012	–0.074	–0.030										
8	Amount of technology use	−	2.42	0.70	−0.196**	–0.021	0.037	−0.147**	–0.075	0.002	0.474**									
9	Past tech use for business/school	−	1.92	1.18	0.353**	−0.165**	−0.194**	0.233**	−0.158**	−0.113*	0.183**	0.056								
10	Frequency tech use for business/school	−	2.40	1.35	0.181**	−0.100*	–0.027	0.096*	–0.049	–0.002	0.116*	0.099*	0.562**							
11	Social support	0.89	5.53	0.96	0.115*	0.077	–0.039	–0.003	0.013	0.022	0.177**	0.162**	0.038	–0.014						
12	Loneliness	0.93	2.80	1.08	−0.249**	0.052	0.025	–0.085	0.034	0.006	−0.164**	–0.003	–0.078	–0.022	−0.507**					
13	State boredom	0.95	3.79	1.16	−0.367**	0.198**	0.114*	−0.145**	0.037	–0.011	−0.136**	0.078	−0.145**	−0.110*	−0.245**	0.617**				
14	State irritability	0.90	3.50	1.31	−0.399**	0.242**	0.117*	−0.140**	0.164**	0.030	−0.129**	0.089	−0.168**	–0.059	−0.250**	0.503**	0.685**			
15	State anger	0.90	2.65	1.23	−0.330**	0.196**	0.094*	–0.078	0.074	0.030	–0.072	0.091*	−0.102*	–0.059	−0.248**	0.502**	0.657**	0.733**		
16	State anxiety	0.84	4.48	1.23	−0.195**	0.301**	–0.024	–0.032	0.075	–0.023	−0.114*	0.041	–0.090	–0.063	–0.080	0.349**	0.571**	0.567**	0.565**	
17	Belongingness	0.80	4.53	1.01	0.187**	0.128**	–0.003	0.004	0.019	0.015	0.091	0.125**	0.056	0.029	0.428**	−0.311**	−0.230**	−0.223**	−0.213**	–0.039

Gender was coded 1 = males and 2 = females. *N* = 463; *p < 0.05; **p < 0.01.

As expected, the frequency of technology use during the lockdown was positively associated with perceived social support. The latter was negatively associated with feelings of loneliness, boredom, anger, and irritability, whereas it was positively associated with perceived belongingness.

A strong correlation (*r* = 0.73) between anger and irritability emerged. In this regard, Vidal-Ribas et al. (2016) stated that “irritability is a mood, and anger is its defining emotion” (p. 557), suggesting that these are different constructs that nevertheless often overlap. In light of the large correlation between the two measures, a composite index for anger/irritability was computed to be used in the following analyses.

Correlational analysis also yielded significant associations between participants’ age and the outcome variables. All these variables, except loneliness, also emerged as significantly associated with participants’ gender. Therefore, multiple regression analyses were conducted for exploring the effects of individual differences and situational variables on feelings of loneliness, irritability, boredom, anger/irritability, anxiety, belongingness, and perceived social support during the lockdown. Overall, significant effects of age and gender consistently emerged in most of the considered variables (see Table 3 for significant results). Hence, they were treated as covariates in all the analyses reported below.

**TABLE 3:** Significant results of simple and multiple linear regressions.

**Predictor**	**Dependent variable**	**Model statistics**	***B***	***SE B***	***β***	**95%CI**		***p***
						***LL***	***UL***	
Age	Social support	*R*^2^ = 0.062, *F*_(9, 453)_ = 3.31, *p* < 0.001**	0.010	0.004	0.135	0.002	0.017	=0.011
Gender		**	0.267	0.103	0.121	0.064	0.469	=0.010
Past technology use		**	0.310	0.082	0.179	0.150	0.471	<0.001
Age	Loneliness	*R*^2^ = 0.081, *F*_(9, 453)_ = 4.44, *p* < 0.001**	–0.020	0.004	–0.246	–0.029	–0.012	<0.001
Past technology use			–0.257	0.091	–0.131	–0.437	–0.078	=0.005
Age	Boredom	*R*^2^ = 0.166, *F*_(9, 453)_ = 10.04, *p* < 0.001**	–0.031	0.004	–0.352	–0.040	–0.022	<0.001
Gender			0.349	0.118	0.131	0.108	0.581	=0.003
Age	Anger/irritability	*R*^2^ = 0.179, *F*_(9, 453)_ = 10.99, *p* < 0.001**	–0.033	0.004	–0.365	–0.041	–0.024	<0.001
Gender			0.470	0.118	0.174	0.238	0.702	<0.001
Age	Anxiety	*R*^2^ = 0.124, *F*_(9, 453)_ = 7.11, *p* < 0.001**	–0.014	0.005	–0.155	–0.024	–0.005	=0.003
Gender			0.777	0.128	0.275	0.525	1.028	<0.001
Age	Belongingness	*R*^2^ = 0.077, *F*_(9, 453)_ = 4.203, *p* < 0.001**	0.019	0.004	0.243	0.011	0.027	<0.001
Gender			0.406	0.108	0.174	0.193	0.619	<0.001

*N = 463; Predictors: (Constant), Age, Gender, Days of isolation, Number of exits, Number of persons living with, House sqm, Past technology use, Past tech use for business/school, Frequency tech use for business/school. LL, lower limit; UL, upper limit.*

### Direct and Indirect Effects

To further explore the associations between the constructs, indirect effects were evaluated considering the joint significance of the components (Yzerbyt et al., 2018) and the bootstrap confidence intervals computed using the PROCESS macro for SPSS (version 3.4, model 4, 5,000 iterations) (Hayes, 2017). Because of the multiple testing, we corrected the alpha level of the component tests with a Bonferroni correction and adjusted the confidence intervals accordingly (Dunn, 1961). Given that we tested five indirect effects, which required six components, we set the alpha level for the component tests at 0.008 and computed the 99% confidence intervals.

Each model considered the frequency of technology use as the focal predictor and perceived social support as the mediator. Loneliness, anger/irritability, boredom, anxiety, and belongingness were separately entered as outcome variables, whereas age and gender were included as covariates.

Table 4 reports the results for the tested models. Supporting our hypothesis, the amount of technology use was a significant predictor of perceived social support.

**TABLE 4:** Significant components and direct and indirect effects.

Predictors	Outcome	Components and direct effects	Indirect effect (completely standardized indirect effect)	*R*^2^	Total effect	*R*^2^
Amount of technology use	Social support	*b* = 0.27, SE = 0.06, *β* = 0.20, *t*(459) = 4.27, *p* < 0.001, 99% CI [0.11, 0.43]	−	−	−	0.06
Social support	Loneliness	*b* = -0.56, SE = 0.04, *β* = -0.50, *t*(458) = −12.37, *p* < 0.001, 99%CI [−0.68, −0.45]	IE = −0.15, 99% CI [−0.25, −0.06] (IE = -0.10, 99% CI [−0.16, −0.04])	0.30	*b* = −0.08, SE = 0.07, *β* = −0.05, *t*(459) = −1.15, *p* = 0.25, 99% CI [−0.27, 0.10]	0.06
Amount of technology use		*b* = 0.07, SE = 0.06, *β* = 0.04, *t*(458) = 1.12, *p* = 0.26, 99% CI [−0.09, 0.23]				
Social support	Boredom	*b* = −0.28, SE = 0.05, *β* = −0.23, *t*(458) = −5.43, *p* < 0.001, 99%CI [−0.42, −0.15]	IE = -0.08, 99% CI [−0.14, −0.02] (IE = −0.05, 99% CI [−0.08, −0.01])	0.20	*b* = 0.02, SE = 0.07, *β* = 0.01, *t*(459) = 0.31, *p* = 0.75, 99% CI [−0.16, 0.21]	0.15
Amount of technology use		*b* = 0.10, SE = 0.07, *β* = 0.06, *t*(458) = 1.38, *p* = 0.17, 99% CI [−0.09, 0.28]				
Social support	Anger/irritability	*b* = −0.32, SE = 0.05, *β* = −0.26, *t*(458) = −6.16, *p* < 0.001, 99%CI [−0.45, −0.18]	IE = −0.09, 99% CI [−0.16, −0.03] (IE = −0.05, 99% CI [−0.09, −0.02])	0.24	*b* = 0.05, SE = 0.07, *β* = 0.03, *t*(459) = 0.70, *p* = 0.48, 99% CI [−0.14, 0.24]	0.17
Amount of technology use		*b* = 0.14, SE = 0.07, *β* = 0.08, *t*(458) = 1.92, *p* = 0.055, 99% CI [−0.05, 0.32]				
Social support	Anxiety	*b* = −0.12, SE = 0.06, *β* = −0.09, *t*(458) = −2.07, *p* = 0.04, 99%CI [−0.27, 0.03]	IE = −0.03, 99% CI [−0.09, 0.006] (IE = −0.02, 99% CI [−0.05, 0.004])	0.12	*b* = 0.03, SE = 0.08, *β* = 0.02, *t*(459) = 0.40, *p* = 0.69, 99% CI [−0.17, 0.24]	0.11
Amount of technology use		*b* = 0.06, SE = 0.08, *β* = 0.04, *t*(458) = 0.80, *p* = 0.42, 99% CI [−0.14, 0.27]				
Social support	Belongingness	*b* = 0.40, SE = 0.04, *β* = 0.38, *t*(458) = 8.98, *p* < 0.001, 99% CI [0.29, 0.52]	IE = 0.11, 99% CI [0.04, 0.20] (IE = 0.07, 99% CI [0.03, 0.13])	0.23	*b* = 0.26, SE = 0.07, *β* = 0.18, *t*(459) = 3.91, *p* < 0.0001, 99% CI [0.09, 0.43]	0.09
Amount of technology use		*b* = 0.15, SE = 0.06, *β* = 0.10, *t*(458) = 2.40, *p* < 0.02, 99% CI [−0.01, 0.31]				

**N* = 463. Statistical analyses were carried out considering gender and age as covariates. See Supplementary Material for the complete results. *IE*, indirect effect.*

**FIGURE 1:**
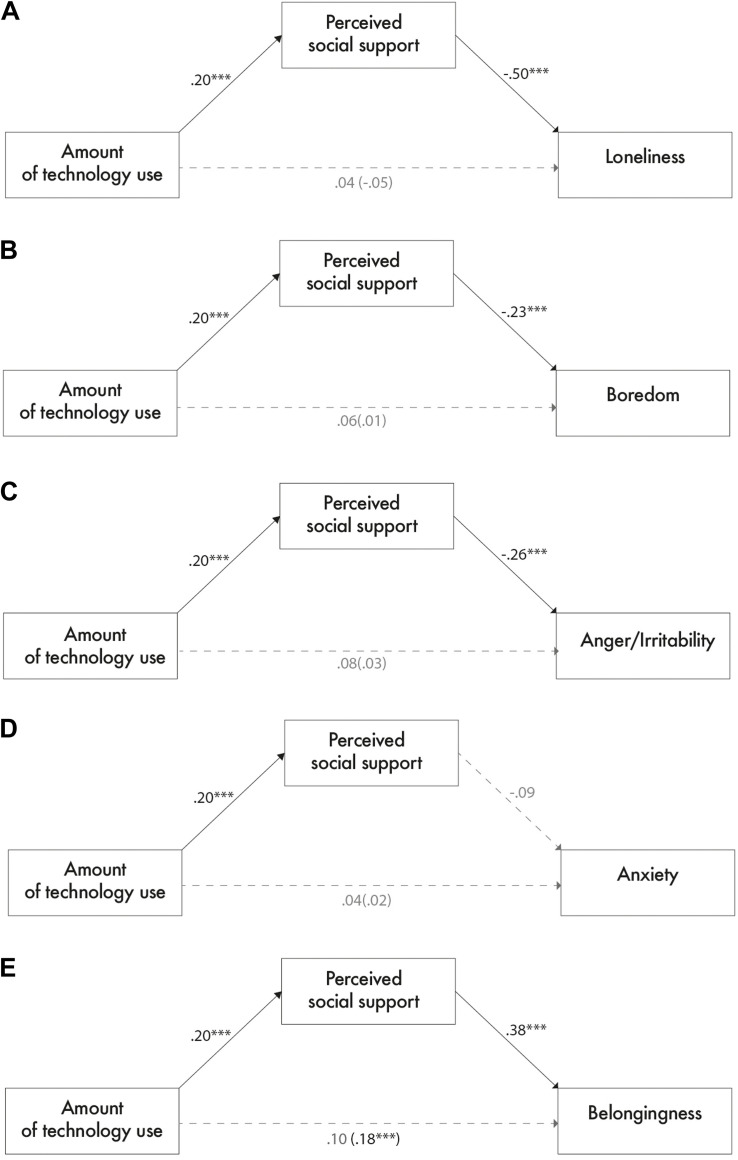
Standardized regression coefficients for the indirect effects of technology usage during the lockdown on loneliness **(A)**, boredom **(B)**, anger and irritability **(C)**, anxiety **(D)**, and belongingness **(E)** through perceived social support. The total effect is in parentheses. ***p < 0.001.

Moreover, perceived social support was negatively associated with loneliness, boredom, and anger/irritability. As expected, it was positively associated with belongingness. Crucially, the proposed theoretical model was sustained by the significance of the indirect effect of technology use *via* social support on these variables (no significant direct effects emerged). Contrary to our hypotheses, no significant effects were found for anxiety (see Figure 1).

## Discussion

The data collected during the lockdown in Italy showed the role of digital technology for maintaining social relations in attenuating the negative consequences of the social distancing imposed to reduce the spread of COVID-19. Specifically, digital technologies for communication and virtual meetings can promote a higher perception of social support, which in turn is associated with lower feelings of loneliness, boredom, and anger/irritability and a greater sense of belonging.

Anxiety was the only variable not affected by the use of digital technologies *via* social support. In this regard, we speculate that the uncertainty generated by a new and unpredictable situation, such as the current pandemic, might have fostered high anxiety levels in people. In effect, worries about health and safety, uncertainty about the future, and no clear perspective about the end of lockdown may have contributed to the maintenance of generalized anxiety among individuals. The social support deriving from the use of technology likely was not sufficient to reduce such high anxiety. Alternatively, sharing fears, predictions, and information about the pandemic could have increased both the perception of social support and anxiety. These influences could have zeroed each other out, resulting in no effect.

Our data also showed that both age and gender were directly associated with the considered constructs (see Supplementary Material). With regard to age, the older the participants were, the less they felt lonely, angry/irritable, bored, and anxious during lockdown. This is not surprising since previous evidence suggested that adolescence is the peak age for experiencing loneliness (see Yang and Victor, 2011 for a review), while other studies showed that older individuals are usually less prone to experience boredom (Vodanovich and Kass, 1990), report more inner control of anger (Phillips et al., 2006), and are generally more capable of adaptive emotion regulation strategies (Orgeta, 2009; Zimmermann and Iwanski, 2014).

Regarding gender, women reported higher levels of psychological distress (i.e., greater feelings of anger/irritability, boredom, and anxiety). This could be due to the fact that, during the pandemic, women had to fulfill more roles compared to men (e.g., caregivers, professional, teacher, and mother), being a group more vulnerable and more at risk in this situation of psychological overload (see also González-Sanguino et al., 2020). Worth mentioning, however, is that our sample was unbalanced (75.1% females).

It is worth mentioning that the association of the amount of technology usage with the perception of social support is small in size (*R*^2^ = 0.06), and the mitigating effects on the considered affective states could be explained mainly by the role of social support. In this regard, technologies are only one resource people can use to experience social support during a lockdown. Some studies show that social support can come from various sources, including religion and community ties (Taylor, 2011). Thus, the use of technology may explain only a reduced part of the variance in social support perception. Additionally, we did not investigate all the possible technologies that people used during the lockdown. Future studies should focus more on the specificity of certain technologies in promoting the perception of social support, such as modern social media, live streaming rooms, and collaborative webinars. It would also be interesting to test whether different technologies can favor different types of social support (informational, instrumental, and emotional support; see Taylor, 2011). In regard with this matter, we speculate that the use of communication technologies may have fostered informational support and mutual help to understand better all the information given during lockdown and locating what resources and coping strategies were needed. Replacing face-to-face relationships with virtual interactions may also have fostered greater emotional support, reassuring people about the uncertainty caused by the ongoing pandemic (see Taylor, 2011).

There are some limitations. First, our data were collected at the beginning of the pandemic and mainly in an area severely affected by the spread of the virus (i.e., Lombardy, Italy). It is possible that in areas less affected or with fewer restrictions, the use of digital technologies to compensate for the lack of social relationships may be weaker. Besides, our results rely on a single correlational study, preventing from drawing any conclusions on the causality between the considered constructs. Thus, future studies should consider a longitudinal or experimental design to test further whether the effects of social isolation can be mitigated by adopting digital technologies, even for longer periods. Second, the measure concerning the amount of digital communication relied on self-report data. Even if recent works suggested that the estimated time spent using a technology (e.g., smartphone) may be an adequate measure of the frequency of use when small resolution of data is required (Andrews et al., 2015), other studies reported that, usually, people underestimate technology usage time by 40% (Lee et al., 2017). Third, we have considered only some of the possible psychological consequences of a lockdown. Indeed, both the World Health Organization (2020b) and American Psychological Association (2020) reported further outcomes, such as depression and posttraumatic stress disorder. Thus, to get a complete picture, future studies should consider a wider number of negative consequences. Moreover, among the possible positive affective states, only belongingness was considered. Future studies should focus more on other possible positive outcomes of using technologies when dealing with social distancing situations. Finally, the present results could have been influenced by the participants’ self-selection. Those who responded to the questionnaire did so starting from a digital link, and therefore, our participants could be already used to communicate adopting digital tools.

## Conclusion

Although the measure of lockdown is proving effective in containing the virus, Brooks et al. (2020) highlighted that the reduction of face-to-face interactions, the loss of freedom, and uncertainty lead to dramatic psychological effects.

In the present study, we showed that using digital technologies for communications and virtual meetings could represent a supportive tool to manage the negative consequences of the social distancing imposed during the COVID-19 outbreak in Italy by assisting social support. As suggested by Waytz and Gray (2018), online communications can improve social relationships, especially when close off-line relationships are not available, such as during an ongoing lockdown. The authors claimed that digital communications can have positive effects, allowing people to empathize with socially distant individuals, fostering emotional and informational support (Taylor, 2011). Nevertheless, all this requires people to be online and connected to technology. These technological solutions are less available to those already at a higher risk of infection, such as the elderly, ill people, and those living in poverty. The lack of reliable access to online services may, therefore, represent an additional burden for those with less access to material and social resources to buffer the negative effects of the coronavirus lockdown. Thus, policymakers should consider implementing strategies to reduce the digital divide in the near future, offering affordable access to communication technologies.

A continued pattern of social distancing, beyond the containment strategy to reduce the spread of the virus, could have broader societal effects, particularly for the most vulnerable (Leigh-Hunt et al., 2017). During the ongoing pandemic, instead of being what the sociologist Sherry Turkle has termed “alone together,” we have access to digital tools that previous generations could not have imagined, and we can now invent new and socially meaningful ways of being together apart.

## Data Availability Statement

The dataset for this study is available through the Open Science Framework (https://osf.io/6g89a/?view_only=1a19465ac45f4256956010271cd18523). The design and analysis plans were not preregistered.

## Ethics Statement

The study was conducted after receiving the ethical approval from the local Commission of the Department of Psychology for minimal risk study. All procedures performed in the study were in accordance with the APA ethical guidelines, the ethical principle of the Helsinki Declaration, and the Oviedo Convention on men’s rights and biomedicine. Full informed consent was obtained before participants started the studies. At the beginning of the survey, the participants were informed about how the data were collected, processed, and stored. They were informed that the estimated duration of the questionnaire was about 15 min. No financial or material incentives were offered to the participants, who took part in the study on a voluntary basis.

## Author Contributions

AG, CB, RV, MD, and FD contributed to the conception and the design of the work. AG was responsible for the data collection and wrote the manuscript with valuable inputs from the remaining authors. AG and MG were responsible for the analysis. All the authors contributed to the interpretation of data and agreed for all aspects of the work and approved the version to be published.

## Conflict of Interest

The authors declare that the research was conducted in the absence of any commercial or financial relationships that could be construed as a potential conflict of interest.
