# Corrigendum: Together Apart: The Mitigating Role of Digital Communication Technologies on Negative Affect During the COVID-19 Outbreak in Italy

**DOI:** 10.3389/fpsyg.2021.701130

**Published:** 2021-05-26

**Authors:** Alessandro Gabbiadini, Cristina Baldissarri, Federica Durante, Roberta Rosa Valtorta, Maria De Rosa, Marcello Gallucci

**Affiliations:** ^1^University of Milano-Bicocca, Department of Psychology – Mind and Behavior Technological Center, Milan, Italy; ^2^University of Milano-Bicocca, Department of Psychology, Milan, Italy

**Keywords:** COVID-19, social isolation, social support, digital technology, negative affect

Due to a clerical error, the values for the amount of technology use for business/school motives before the lockdown were reported incorrectly in Table 2 and Table 3. The amended Table 2 and Table 3 appears below.

**Table 2:** Descriptive statistics and correlations among variables.

		**α**	***M***	**SD**	**1**	**2**	**3**	**4**	**5**	**6**	**7**	**8**	**9**	**10**	**11**	**12**	**13**	**14**	**15**	**16**
1	Age	–	31.26	13.19																
2	Gender	–	–	–	−0.163^**^															
3	Days of isolation	–	14.15	7.18	−0.206^**^	0.075														
4	Number of exits	–	2.36	1.63	0.358^**^	−0.089	−0.550^**^													
5	Number of persons living with	–	2.96	1.30	−0.283^**^	0.027	0.075	−0.118^*^												
6	House sqm	–	123.09	77.09	−0.106^*^	0.002	0.085	−0.055	0.357^**^											
7	Past technology use	–	1.75	0.55	0.168^**^	−0.149^**^	−0.013	0.012	−0.074	−0.030										
8	Amount of technology use	–	2.42	0.70	−0.196^**^	−0.021	0.037	−0.147^**^	−0.075	0.002	0.474^**^									
9	Past tech use for business/school	–	1.92	1.18	0.353^**^	−0.165^**^	−0.194^**^	0.233^**^	−0.158^**^	−0.113^*^	0.183^**^	0.056								
10	Frequency tech use for business/school	–	2.40	1.35	0.181^**^	−0.100^*^	−0.027	0.096^*^	−0.049	−0.002	0.116^*^	0.099^*^	0.562^**^							
11	Social support	0.89	5.53	0.96	0.115^*^	0.077	−0.039	−0.003	0.013	0.022	0.177^**^	0.162^**^	0.038	−0.014						
12	Loneliness	0.93	2.80	1.08	−0.249^**^	0.052	0.025	−0.085	0.034	0.006	−0.164^**^	−0.003	−0.078	−0.022	−0.507^**^					
13	State boredom	0.95	3.79	1.16	−0.367^**^	0.198^**^	0.114^*^	−0.145^**^	0.037	−0.011	−0.136^**^	0.078	−0.145^**^	−0.110^*^	−0.245^**^	0.617^**^				
14	State irritability	0.90	3.50	1.31	−0.399^**^	0.242^**^	0.117^*^	−0.140^**^	0.164^**^	0.030	−0.129^**^	0.089	−0.168^**^	−0.059	−0.250^**^	0.503^**^	0.685^**^			
15	State anger	0.90	2.65	1.23	−0.330^**^	0.196^**^	0.094^*^	−0.078	0.074	0.030	−0.072	0.091^*^	−0.102^*^	−0.059	−0.248^**^	0.502^**^	0.657^**^	0.733^**^		
16	State anxiety	0.84	4.48	1.23	−0.195^**^	0.301^**^	−0.024	−0.032	0.075	−0.023	−0.114^*^	0.041	−0.090	−0.063	−0.080	0.349^**^	0.571^**^	0.567^**^	0.565^**^	
17	Belongingness	0.80	4.53	1.01	0.187^**^	0.128^**^	−0.003	0.004	0.019	0.015	0.091	0.125^**^	0.056	0.029	0.428^**^	−0.311^**^	−0.230^**^	−0.223^**^	−0.213^**^	-0.039

*Gender was coded 1 = males and 2 = females. N = 463*;

* *p < 0.05*;

** *p < 0.01*.

**Table 3:** Significant results of simple and multiple linear regressions.

**Predictor**	**Dependent variable**	**Model statistics**	***B***	***SE B***	**β**	**95%CI**		***p***
						***LL***	***UL***	
Age	Social support	*R^2^ =* 0.062, *F*_(9, 453)_ = 3.31, *p* < 0.001	0.010	0.004	0.135	0.002	0.017	=0.011
Gender			0.267	0.103	0.121	0.064	0.469	=0.010
Past technology use			0.310	0.082	0.179	0.150	0.471	<0.001
Age	Loneliness	*R^2^ =* 0.081, *F*_(9, 453)_ = 4.44, *p* < 0.001	−0.020	0.004	−0.246	−0.029	−0.012	<0.001
Past technology use			−0.257	0.091	−0.131	−0.437	−0.078	=0.005
Age	Boredom	*R^2^ =* 0.166, *F*_(9, 453)_ = 10.04, *p* < 0.001	−0.031	0.004	−0.352	−0.040	−0.022	<0.001
Gender			0.349	0.118	0.131	0.108	0.581	=0.003
Age	Anger/irritability	*R^2^ =* 0.179, *F*_(9, 453)_ = 10.99, *p* < 0.001	−0.033	0.004	−0.365	−0.041	−0.024	<0.001
Gender			0.470	0.118	0.174	0.238	0.702	<0.001
Age	Anxiety	*R^2^ =* 0.124, *F*_(9, 453)_ = 7.11, *p* < 0.001	−0.014	0.005	−0.155	−0.024	−0.005	=0.003
Gender			0.777	0.128	0.275	0.525	1.028	<0.001
Age	Belongingness	*R^2^ =* 0.077, *F*_(9, 453)_ = 4.203, *p* < 0.001	0.019	0.004	0.243	0.011	0.027	<0.001
Gender			0.406	0.108	0.174	0.193	0.619	<0.001

*N = 463; Predictors: (Constant), Age, Gender, Days of isolation, Number of exits, Number of persons living with, House sqm, Past technology use, Past tech use for business/school, Frequency tech use for business/school. LL, lower limit; UL, upper limit*.

The authors apologize for this error and state that this does not change the scientific conclusions of the article in any way. The original article has been updated.

