# Supplementary material for: Together Apart: The Mitigating Role of Digital Communication Technologies on Negative Affect During the COVID-19 Outbreak in Italy
Source: Front Psychol. 2020 Oct 21;11:554678. doi: 10.3389/fpsyg.2020.554678 (PMC7609360; doi:10.3389/fpsyg.2020.554678)
Supplement: Supplementary file 1 [file Data_Sheet_1.docx]

Supplementary Material

*Together Apart: The Mitigating Role of Digital Communication Technologies on Negative Affect During the COVID-19 Outbreak in Italy*

# Supplementary analyses

## Normality test, outlier and influential cases inspection

Outlier were inspected by plotting Cook’s distances by centered leverage values of the residuals (Cook, 1977) for each regression model. Two influential cases emerged (see Table 1, Table 2, Table 3 and Table 4 for a comparison of results with and without influential cases).

**Table 1.** *Normality tests, Cook’s distances and Centered leverage values for the whole sample*

| Predictors | Outcome | *Residual Skewness* | *Residual Kurtosis* | *Residual Cook’s Distance*  *(Min / Max)* | *Centered leverage value*  *(Min / Max)* | *Influential points* |
| --- | --- | --- | --- | --- | --- | --- |
| Frequency of technology use | Social support | -.663 | .155 | .000 / .045 | .001 / .032 | ID= 524 |
| Social support | Loneliness | .520 | .223 | .000 / .069 | .001 / .032 | ID = 46 |
| Frequency of technology use |  | .600 | -.005 | .000 / .027 | .001 / .032 | -- |
| Frequency & Social support |  | .535 | .257 | .000 / .056 | .001 / .043 | ID = 46 |
| Social support | Boredom | -.167 | -.328 | .000 / .033 | .001 / .032 | ID = 46 |
| Frequency of technology use |  | -.056 | -.362 | .000 / .031 | .001 / .032 | -- |
| Frequency & Social support |  | -.163 | -.323 | .000 / .027 | .001 / .043 | ID = 46 |
| Social support | Anger/Irritability | .519 | .306 | .000 / .064 | .001 / .032 | -- |
| Frequency of technology use |  | .578 | .276 | .000 / .057 | .001 / .032 | -- |
| Frequency & Social support |  | .532 | .318 | .000 / .051 | .001 / .043 | ID = 46 |
| Social support | Anxiety | -.251 | -.518 | .000 / .033 | .001 / .032 | ID = 46 |
| Frequency of technology use |  | -.281 | -.518 | .000 / .021 | .001 / .032 | -- |
| Frequency & Social support |  | -.245 | -.516 | .000 / .027 | .001 / .043 | ID = 46 |
| Social support | Belongingness | .023 | -.175 | .000 / .032 | .001 / .032 | -- |
| Frequency of technology use |  | -.160 | -.224 | .000 / .019 | .001 / .032 | -- |
| Frequency & Social support |  | -.013 | -.205 | .000 / .043 | .001 / .043 | -- |

Note. *N* = 465. All the analyses were performed considering age and gender as covariates.

**Table 2.** *Normality tests, Cook’s distances and Centered leverage values without influential points*

| Predictors | Outcome | *Residual Skewness* | *Residual Kurtosis* | *Residual Cook’s Distance*  *(Min / Max)* | *Centered leverage value*  *(Min / Max)* |
| --- | --- | --- | --- | --- | --- |
| Frequency of technology use | Social support | -.616 | .016 | .000 / .035 | .001 / .032 |
| Social support | Loneliness | .557 | .195 | .000 / .024 | .001 / .034 |
| Frequency of technology use |  | .597 | -.015 | .000 / .027 | .001 / .032 |
| Frequency & Social support |  | .573 | .218 | .000 / .024 | .001 / .036 |
| Social support | Boredom | -.169 | -.319 | .000 / .027 | .001 / .034 |
| Frequency of technology use |  | -.061 | -.362 | .000 / .031 | .001 / .032 |
| Frequency & Social support |  | -.167 | -.311 | .000 / .024 | .001 / .036 |
| Social support | Anger/Irritability | .533 | .301 | .000 / .064 | .001 / .034 |
| Frequency of technology use |  | .576 | .273 | .000 / .057 | .001 / .032 |
| Frequency & Social support |  | .545 | .313 | .000 / .052 | .001 / .036 |
| Social support | Anxiety | -.247 | -.509 | .000 / .021 | .001 / .034 |
| Frequency of technology use |  | -.280 | -.506 | .000 / .021 | .001 / .032 |
| Frequency & Social support |  | -.241 | -.509 | .000 / .019 | .001 / .036 |
| Social support | Belongingness | .018 | -.173 | .000 / .066 | .001 / .034 |
| Frequency of technology use |  | -.160 | -.215 | .000 / .019 | .001 / .032 |
| Frequency & Social support |  | -.012 | -.201 | .000 / .056 | .001 / .036 |

Note. *N* = 463. All the analyses were performed considering age and gender as covariates.

**Table 3.** *Significant components, direct and indirect effects with covariates*

| Predictors | Outcome | Components and direct effects | Indirect effect  (Completely standardized indirect effect) | *R^2^* | Total effect | *R^2^* |
| --- | --- | --- | --- | --- | --- | --- |
| Amount of technology use | Social support | *b*=.27, *SE*=.06, *β*=.20, t(459)=4.27, *p*<.001, 99%CI[0.11, 0.43] | - | - | - | .06 |
| Age |  | *b*=.01, *SE*=.00, *β*=.17, t(459)=3.65, *p<*.001, 99%CI[0.004, 0.02] |  |  |  |  |
| Gender |  | *b*=.24, *SE*=.10, *β*=.11, t(459)=2.38, *p*=.018, 99%CI[-0.02, 0.50] |  |  |  |  |
| Social support | Loneliness | *b*=-.56, *SE*=.04, *β*=−.50, t(458)=-12.37, *p*<.001, 99%CI[-0.68, -0.45] | *IE=*-.15, 99%CI [-0.25, -0.06]  *(IE=*-.10, 99%CI [-0.16, -0.04]) | .30 | *b*=-.08, *SE*=.07, *β*=−.05, t(459)=-1.15, *p=*.25, 99%CI[-0.27, 0.10] | .06 |
| Amount of technology use |  | *b*=.07, *SE*=.06, *β*=. 04, t(458)=1.12, *p=*.26, 99%CI[-0.09, 0.23] |  |  |  |  |
| Age |  | *b*=-.01, *SE*=.00, *β*=−.17, t(458)=-4.19, *p*<.001, 99%CI[-0.02, -0.005] |  |  |  |  |
| Gender |  | *b*=.16, *SE*=.10, *β*=.06, t(458)=1.58, *p=*.11, 99%CI[-0.10, 0.41] |  |  |  |  |
| Social support | Boredom | *b*=-.28, *SE*=.05, *β*=−.23, t(458)=-5.43, *p*<.001, 99%CI[-0.42, -0.15] | *IE*=-.08, 99%CI [-0.14, -0.02]  *(IE=*-.05, 99%CI [-0.08, -0.01]) | .20 | *b*=.02, *SE*=.07, *β*=.01, t(459)=0.31, *p=*.75, 99%CI[-0.16, 0.21] | .15 |
| Amount of technology use |  | *b*=.10, *SE*=.07, *β*=.06, t(458)=1.38, *p=*.17, 99%CI[-0.09, 0.28] |  |  |  |  |
| Age |  | *b*=-.03, *SE*=.00, *β*=−.30, t(458)=-6.89, *p*<.001, 99%CI[-0.04, -0.02] |  |  |  |  |
| Gender |  | *b*=.45, *SE*=.11, *β*=.17, t(458)=3.95, *p*<.001, 99%CI[0.15, 0.74] |  |  |  |  |
| Social support | Anger/irritability | *b*=-.32, *SE*=.05, *β*=−.26, t(458)=-6.16, *p*<.001, 99%CI[-0.45, -0.18] | *IE*=-.09, 99% CI [-0.16, -0.03]  *(IE=*-.05, 99%CI [-0.09, -0.02]) | .24 | *b*=.05, *SE*=.07, *β*=.03, t(459)=0.70, *p=*.48, 99%CI[-0.14, 0.24] | .17 |
| Amount of technology use |  | *b*=.14, *SE*=.07, *β*=.08, t(458)=1.92, *p=*.055, 99%CI[-0.05, 0.32] |  |  |  |  |
| Age |  | *b*=-.03, *SE*=.00, *β*=−.30, t(458)=-7.03, *p*<.001, 99%CI[-0.04, -0.02] |  |  |  |  |
| Gender |  | *b*=.55, *SE*=.11, *β*=.20, t(458)=4.90, *p*<.001, 99%CI[0.26, 0.84] |  |  |  |  |
| Social support | Anxiety | *b*=-.12, *SE*=.06, *β*=−.09, t(458)=-2.07, *p=*.04, 99%CI[-0.27, 0.03] | *IE*= -.03, 99%CI [-0.09, 0.006]  *(IE=*-.02, 99%CI [-0.05, 0.004]) | .12 | *b*=.03, *SE*=.08, *β*=.02, t(459)=0.40, *p=*.69, 99%CI[-0.17, 0.24] | .11 |
| Amount of technology use |  | *b*=.06, *SE*=.08, *β*=.04, t(458)=0.80, *p=*.42, 99%CI[-0.14, 0.27] |  |  |  |  |
| Age |  | *b*=-.01, *SE*=.00, *β*=−.13, t(458)=-2.83, *p<*.005, 99%CI[-0.02, -0.001] |  |  |  |  |
| Gender |  | *b*=.81, *SE*=.13, *β*=.29, t(458)=6.44, *p*<.001, 99%CI[0.49, 1.14] |  |  |  |  |
| Social support | Belongingness | *b*=.40, *SE*=.04, *β*=.38, t(458)=8.98, *p*<.001, 99%CI[0.29, 0.52] | *IE*= .11, 99%CI [0.04, 0.20]  *(IE=*.07, 99%CI [0.03, 0.13]) | .23 | *b*=.26, *SE*=.07, *β*=.18, t(459)=3.91, *p*<.001, 99%CI[0.09, 0.43] | .09 |
| Amount of technology use |  | *b*=.15, *SE*=.06, *β*=.10, t(458)=2.40, *p*<.02, 99%CI[-0.01, 0.31] |  |  |  |  |
| Age |  | *b*=.01, *SE*=.00, *β*=.18, t(458)=4.28, *p*<.001, 99%CI[0.006, 0.02] |  |  |  |  |
| Gender |  | *b*=.30, *SE*=.10, *β*=.13, t(458)=3.12, *p*<.002, 99%CI[0.05, 0.56] |  |  |  |  |

Note. *N*=463. *IE* = Indirect Effect

**Table 4.** *Significant components, direct and indirect effects with covariates when considering the influential data points*

| Predictors | Outcome | Components and direct effects | Indirect effect  (Completely standardized indirect effect) | *R^2^* | Total effect | *R^2^* |
| --- | --- | --- | --- | --- | --- | --- |
| Amount of technology use | Social support | *b*=.25, *SE*=.06, *β*=.18, t(461)=3.92, *p*<.001, 99%CI[0.08, 0.41] | - | - | - | .05 |
| Age |  | *b*=.01, *SE*=.00, *β*=.16, t(461)=3.44, *p<*.001, 99%CI[0.003, 0.02] |  |  |  |  |
| Gender |  | *b*=.26, *SE*=.10, *β*=.12, t(461)=2.53, *p*=.012, 99%CI[-0.005, 0.52] |  |  |  |  |
| Social support | Loneliness | *b*=-.53, *SE*=.04, *β*=−.48, t(460)=-12.37, *p*<.001, 99%CI[-0.68, -0.44] | *IE=*-.13, 99%CI [-0.23, -0.04]  *(IE=*-.08., 99%CI [-0.14, -0.03]) | .28 | *b*=-.08, *SE*=.07, *β*=−.05, t(461)=-1.14, *p=*.25, 99%CI[-0.26, 0.10] | .06 |
| Amount of technology use |  | *b*=.05, *SE*=.06, *β*=. 04, t(460)=0.85, *p=*.39, 99%CI[-0.10, 0.21] |  |  |  |  |
| Age |  | *b*=-.01, *SE*=.00, *β*=−.18, t(460)=-4.35, *p*<.001, 99%CI[-0.02, -0.006] |  |  |  |  |
| Gender |  | *b*=.16, *SE*=.10, *β*=.06, t(460)=1.68, *p=*.09, 99%CI[-0.09, 0.42] |  |  |  |  |
| Social support | Boredom | *b*=-.26, *SE*=.05, *β*=−.22, t(460)=-5.10, *p*<.001, 99%CI[-0.39, -0.12] | *IE*=-.06, 99%CI [-0.13, -0.01]  *(IE=*-.04, 99%CI [-0.08, -0.01]) | .20 | *b*=.02, *SE*=.07, *β*=.01, t(461)=0.28, *p=*.78, 99%CI[-0.16, 0.20] | .15 |
| Amount of technology use |  | *b*=.08, *SE*=.07, *β*=.05, t(460)=1.20, *p=*.23, 99%CI[-0.09, 0.27] |  |  |  |  |
| Age |  | *b*=-.02, *SE*=.00, *β*=−.30, t(460)=-7.03, *p*<.001, 99%CI[-0.03, -0.01] |  |  |  |  |
| Gender |  | *b*=.45, *SE*=.11, *β*=.17, t(460)=4.03, *p*<.001, 99%CI[0.16, 0.75] |  |  |  |  |
| Social support | Anger/irritability | *b*=-.29, *SE*=.05, *β*=−.24, t(460)=-5.82, *p*<.001, 99%CI[-0.42, -0.16] | *IE*=-.07, 99% CI [-0.14, -0.02]  *(IE=*-.04, 99%CI [-0.08, -0.01]) | .23 | *b*=.05, *SE*=.07, *β*=.03, t(461)=0.73, *p=*.46, 99%CI[-0.13, 0.23] | .17 |
| Amount of technology use |  | *b*=.12, *SE*=.07, *β*=.07, t(460)=1.79, *p=*.07, 99%CI[-0.05, 0.30] |  |  |  |  |
| Age |  | *b*=-.03, *SE*=.00, *β*=−.30, t(460)=-7.41, *p*<.001, 99%CI[-0.03, -0.01] |  |  |  |  |
| Gender |  | *b*=.56, *SE*=.11, *β*=.21, t(460)=5.00, *p*<.001, 99%CI[0.27, 0.85] |  |  |  |  |
| Social support | Anxiety | *b*=-.10, *SE*=.05, *β*=−.08, t(460)=-1.85, *p=*.06, 99%CI[-0.25, 0.04] | *IE*= -.02, 99%CI [-0.08, 0.008]  *(IE=*-.01, 99%CI [-0.04, 0.004]) | .12 | *b*=.04, *SE*=.07, *β*=.02, t(461)=0.57, *p=*.56, 99%CI[-0.15, 0.24] | .11 |
| Amount of technology use |  | *b*=.07, *SE*=.08, *β*=.04, t(460)=0.90, *p=*.37, 99%CI[-0.13, 0.27] |  |  |  |  |
| Age |  | *b*=-.01, *SE*=.00, *β*=−.13, t(460)=-2.78, *p<*.006, 99%CI[-0.02, -0.0008] |  |  |  |  |
| Gender |  | *b*=.83, *SE*=.12, *β*=.29, t(460)=6.60, *p*<.001, 99%CI[0.50, 1.16] |  |  |  |  |
| Social support | Belongingness | *b*=.40, *SE*=.04, *β*=.38, t(460)=9.01, *p*<.001, 99%CI[0.28, 0.51] | *IE*= .10, 99%CI [.02, 0.18]  *(IE=*.07, 99%CI [0.02, 0.12]) | .23 | *b*=.26, *SE*=.06, *β*=.18, t(461)=4.06, *p*<.001, 99%CI[0.9, 0.43] | .09 |
| Amount of technology use |  | *b*=.17, *SE*=.06, *β*=.11, t(460)=2.71, *p*<.007, 99%CI[0.008, 0.32] |  |  |  |  |
| Age |  | *b*=.01, *SE*=.00, *β*=.19, t(460)=4.48, *p*<.001, 99%CI[0.006, 0.02] |  |  |  |  |
| Gender |  | *b*=.31, *SE*=.10, *β*=.13, t(460)=3.23, *p*<.002, 99%CI[0.06, 0.56] |  |  |  |  |

Note. *N*=465. *IE* = Indirect Effect

## Source of social support

We explored whether the use of digital technologies during the lockdown was associated with a particular source of social support (i.e., family, friends, significant others), especially within the context of a lockdown.

Thus, three different indexes for sources of social support were computed (support from the family, 4 items, α = .89; support from friends, 4 items, α = .90; support from significant others, 4 items, α = .92). A set of simple linear regressions was then run to predict different type of social support from the amount of technology use (gender and age were entered as covariates). Tech usage significantly predicted social support from the family, *b* = .22, β = .12, *t*(459) = 2.58, *p* = .010, from friends, *b* =.26, β = .16, *t*(459) = 3.49, *p* = .001, and from significant others, *b* = .32, β = .16, *t*(459) = 3.53, *p* < .001. These results suggest that the amount of technology usage during the lockdown was associated with all three sources of social support tapped by the adopted scale.

## Post-hoc power analysis

Post-hoc power analyses for each tested model was conducted using the Monte Carlo Power Analysis for Indirect Effects (<https://schoemanna.shinyapps.io/mc_power_med/>) developed by Schoemann, Boulton, and Short (2017), considering *N* = 463, input method = “standardized coefficients”, 1.000 replications, 20.000 Monte Carlo Draws and a 95% confidence level. A comparison of the power obtained for each tested model with a model considering small indirect effects (i.e., IE = .20) is reported in Table 5.

**Table 5.** *Post-hoc power analyses for the tested models compared with a model with small indirect effects*

|  | Predictor | Mediator | Outcome | Power for small effects  (IE = .10) | Actual Power |
| --- | --- | --- | --- | --- | --- |
| Model 1 | Technology use | Social support | Loneliness | .98 | .99 |
| Model 2 | Technology use | Social support | State Boredom | .98 | .99 |
| Model 3 | Technology use | Social support | Anger/Irritability | . 98 | .99 |
| Model 4 | Technology use | Social support | State Anxiety | . 98 | .48 |
| Model 5 | Technology use | Social support | Belongingness | . 98 | .99 |

*Note*. Power for all models was tested considering age and gender as covariates.
